# Tactile perception of pleasantness in relation to perceived softness

**DOI:** 10.1038/s41598-020-68034-x

**Published:** 2020-07-07

**Authors:** Achille Pasqualotto, Megan Ng, Zheng Yee Tan, Ryo Kitada

**Affiliations:** 1 School of Psychology, University of Nottingham Malaysia, Semenyih, Malaysia; 2 Division of Psychology, School of Social Sciences, Nanyang Technological University, 48 Nanyang Avenue, Singapore, 639818 Singapore

**Keywords:** Psychology, Somatosensory system

## Abstract

The sense of touch allows us to infer objects’ physical properties, while the same input also produces affective sensations. These affective sensations are important for interpersonal relationships and personal well-being, which raises the possibility that tactile preferences are adapted to the characteristics of the skin. Previous studies examined how physical properties such as surface roughness and temperature influence affective sensations; however, little is known about the effect of compliance (physical correlate of softness) on pleasantness. Thus, we investigated the psychophysical link between *softness* and *pleasantness*. Pieces of human skin-like rubber with different compliances were pressed against participants’ fingers. Two groups of participants numerically estimated the perceived magnitude of either pleasantness or softness. The perceived magnitude of pleasantness and softness both increased monotonically as a function of increasing object compliance, levelling off at around the end of the stimulus range. However, inter-subject variability was greater for pleasantness than for perceived softness, whereas the slope of the linear function fit to the magnitude estimates was steeper for softness than for pleasantness. These results indicate that object compliance is a critical physical determinant for pleasantness, whereas the effect of compliance on pleasantness was more variable among individuals than the effect on softness was.

## Introduction

When we touch objects, we not only perceive and discriminate their physical properties but also experience associated affective sensations. The former aspect is referred to as discriminative touch, whereas the latter aspect is referred to as affective touch. These two aspects of touch can contribute to a good quality of life and a general sense of well-being^1–4^. For example, interpersonal touch is associated with cognitive and emotional development in humans and animals^5,6^, while also alleviating unpleasantness associated with physical and social pain^7,8^ and maintaining and promoting social structures^9^. The findings of these studies raise the possibility that our tactile preferences are adapted to the characteristics of the skin in order to satisfy the urgent need for social bonds. However, apart from nociception (e.g., itching and pain), the psychophysical attributes of affective touch have been poorly understood.

A number of previous studies have indicated how a unique set of unmyelinated afferent fibres can contribute to the perception of tactile pleasantness. These fibres, called C-tactile fibres, are located in hairy skin and respond vigorously to slow, light stroking against the skin^10^. Stroking ranging from 1 to 10 cm/s provides the most pleasant sensation, which correlates with the firing frequency of C-tactile fibres but not with the frequency of other receptors that are sensitive to mechanical distortion of the skin (mechanoreceptors)^11^. Stroking the forearm and dorsum of the hand with a soft brush can create a pleasant experience for a patient lacking large myelinated afferents^12^. Because slow and light stimulation of the skin can occur in inter-personal interaction, the authors proposed that C-tactile fibres have a particular potential to elicit a pleasant subjective experience during gentle touch between individuals (the “social touch” hypothesis)^13^.

However, the contribution of C-tactile fibres to pleasant touch is limited by the fact that the palmar side of the hand (glabrous skin) does not contain C-tactile fibres, even though touching some objects with our palm evokes pleasantness. It has been proposed that pleasant touch from hairy skin represents an innate non-learned process involving the C-tactile fibres, while pleasant touch from the glabrous skin (innervated by Aβ afferents) represents an analytical process based on previous experience^14^. Nevertheless, the perceptual aspects of pleasant touch in the glabrous skin are still poorly understood.

In contrast to hairy skin, the nature of discriminative touch and mechanoreceptors in the hand have been more heavily investigated. Previous psychophysical studies have shown that physical parameters of objects can change discriminative aspects of tactile perception^15^. Specific mechanoreceptors, or a combination of them, in the hand can account for the tactile perception of object properties^16–21^. For instance, spacing between raised elements of a surface can account for the magnitude of perceived coarse roughness and spatial variation of SA-I activity can account for the magnitude of perceived coarse roughness^16–18,20,21^. Thus, one way to understand the psychophysical nature of affective touch is to examine the extent to which critical parameters previously assessed, with respect to their influence on discriminative touch, influence corresponding affective touch.

Tangible object properties are categorised into macro-geometrical properties, such as shape and orientation, and material properties, such as roughness and softness^15^. Material properties are easier to perceive through touch than macro-geometrical properties^22^. Among material properties, roughness, temperature, and softness are prominent perceptual dimensions of textures^23,24^. One line of research has investigated the relationship between pleasantness and dimensions of textures^25–28^. These studies have demonstrated the psychophysical relationships between affective and discriminative aspects of touch regarding roughness^25–27^ and temperature^28^. However, to our knowledge, no previous study has investigated such a link regarding tactile perception of softness. Another line of research associated ratings of adjectives (e.g., relaxing, boring, and dominant) with a variety of stimuli to determine the factors involved in affective touch^29–31^. Nevertheless, the relationship between softness and pleasantness is still not clear.

Previous studies have examined physical factors that determine tactile perception of softness^15,32–38^ and some have provided two important findings. First, perceived softness increases as a function of object compliance, defined as the magnitude of deformation of an object under applied force. This indicates that compliance is a critical determinant in perceiving softness. Second, the patterns of magnitude estimates of perceived softness were highly similar when compressional force applied to one’s fingers ranged from 0.15 to 3 N^34^. The softness of objects should remain highly similar regardless of how much force is applied when it is touched. Thus, this indicates the perceptual constancy of softness against applied force.

It has been proposed that affective touch is related to how physical contact with an object can potentially influence the physiological condition of the self body^27,39^ and social bonding^9,40^. More specifically, the emotions evoked by contact with an object depend on whether that contact could be either hazardous or beneficial. For example, contact with stiff objects (e.g., iron) is potentially harmful because hitting one’s finger against such an object could cause injury. Softer objects not only decrease the chance of injury but may also be associated with social interactions similar to the caregiver-infant relationship. Given this framework, we would expect pleasantness to increase as a function of object compliance.

On the other hand, veridical perception should be less critical for assessing pleasantness than softness. Unlike softness, which may benefit from the stabilising influence of perceptual constancies during the assessment and manipulation of external objects, affective touch is presumably subject to fewer such demands and influenced by other factors such as people’s own preferences and the way a person interacts with an object^27^. For example, a soft object may feel much more pleasant than a hard object to some people, while other people may perceive only a slight difference between the two. Thus, there may be more individual differences in the effect of compliance on pleasantness than on softness. Moreover, touching a soft pillow can feel pleasant, but slamming the same pillow feels less pleasant. Therefore, the force applied to an object should cause a greater effect on an affective sensation than the perception of softness does. However, to the best of our knowledge, no previous study has examined the effect of compliance on affective sensation.

In the present study, we investigated the psychophysical relationship between softness and pleasantness. Participants were asked to evaluate either how soft or how pleasant an object felt after it was placed against their fingers. We predicted that both perceived pleasantness and softness would increase as a function of compliance, whereas pleasantness would be more strongly affected by individual differences and applied force than perceived softness would be.

## Results

**Figure 1:**
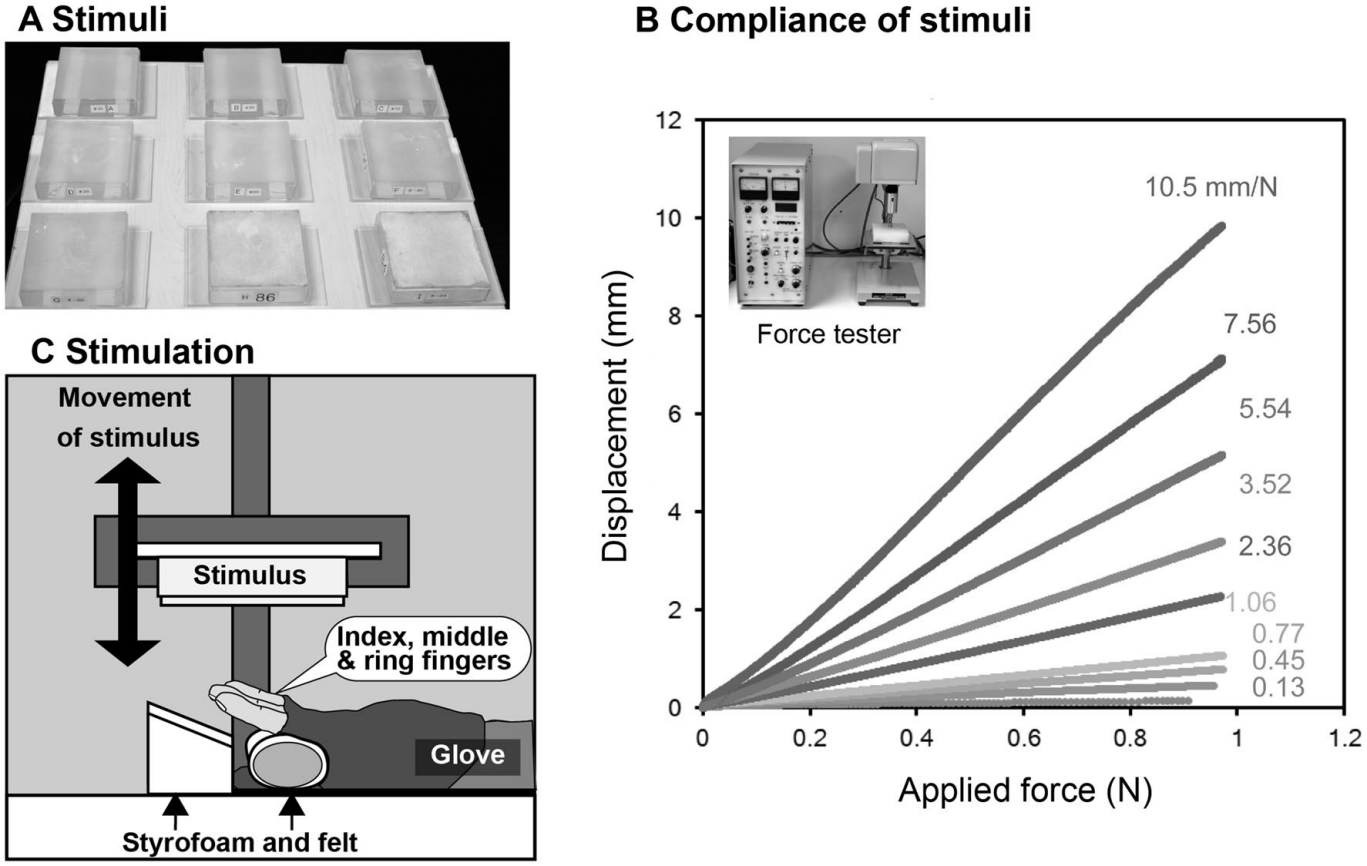
Stimuli and stimulus presentation. **A** We used 9 stimuli made of polyurethane rubbers. To minimise tackiness, baby powder (corn starch) was placed on the surface of the stimuli. **B** Compliance of each stimulus was defined by calculating slopes fit to patterns of displacement as a function of applied force. Value for each line indicates the compliance for each stimulus. **C** Participants used their right hands with their fingers facing upward, supported by Styrofoam and a roll of felt. The customised apparatus moved at 5 cm/s and pushed against the participants’ index, middle, and ring fingers until the force reached the specified maximum value (5 N or 20 N).

In our experiment, a customised stimulator pressed one of nine polyurethane stimuli against participants’ index, middle, and ring fingers (Fig. 1). Compliance of stimuli ranged from 0.13 to 10.53 mm/N. The speed of the stimulation was constant, whereas the maximum force applied to the fingers was either 5 N or 20 N. None of the participants reported experiencing pain during the experiment. We adopted the absolute magnitude estimation procedure^41^, in which participants were instructed to choose a positive number that best represented perceived softness in one group and pleasantness in the other group. The values were analysed using the conventional procedure^21,27^.

### Softness

**Figure 2:**
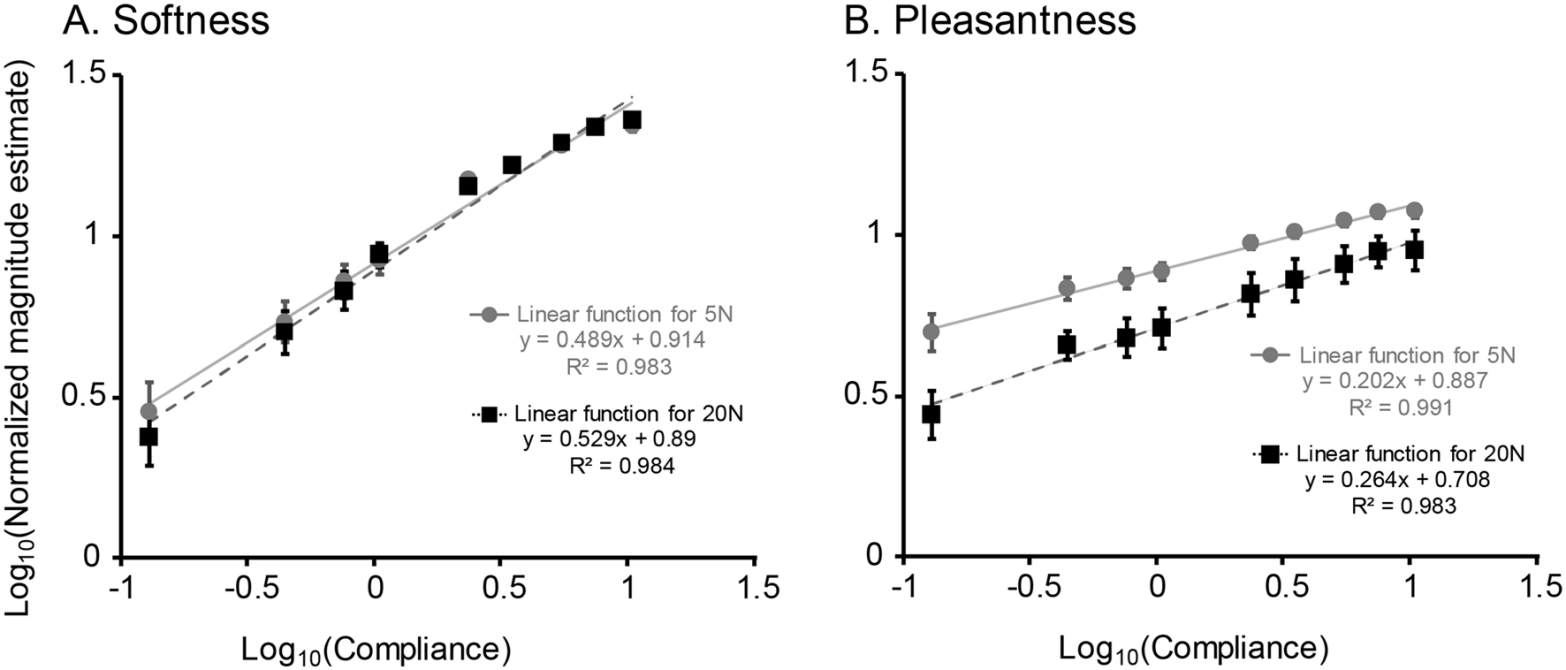
Magnitude estimates of softness and pleasantness. **A** Mean log10 normalised magnitude estimates (ME) of softness as a function of compliance for low and high maximum force. **B** Mean log10 normalised ME of pleasantness as a function of compliance for low and high maximum force. Each data point indicates mean ± SEM of 24 participants (24 for each instruction). Note that two groups of participants evaluated softness and pleasantness separately.

We conducted a two-way ANOVA on magnitude estimates of softness with compliance (9 levels) and maximum force (two levels: 5 N or 20 N) as within-subject variables. The analysis revealed a significant main effect of compliance [*F*(1.3, 29.7) = 64.28, *p* < 0.001, *η*_*p*_^2^ = 0.736]. The same ANOVA showed neither a significant main effect of force [*F*(1, 23) = 1.46, *p* = 0.239, *η*_*p*_^2^ = 0.06] nor significant interaction [*F*(1, 23) = 1.89, *p* = 0.126, *η*_*p*_^2^ = 0.076]. Figure 2A shows softness as a function of compliance on base-10 logarithmic scales for two levels of maximum force. Magnitude estimates monotonically increased with the increase of compliance, whereas estimates tended to level off at 7.56 mm/N (i.e., 0.879 in the horizontal axis in Fig. 2A). We fit a linear function to the magnitude estimates as a function of compliance. R^2^ values (the coefficient of determination) for fitted functions with 5 N and 20 N exceeded 0.98, indicating that the linear trend explained most of the variance in the averaged data.

### Pleasantness

We conducted the same ANOVA as described above on magnitude estimates of pleasantness. This analysis showed a significant main effect of compliance [*F*(1.5, 35.0) = 35.55, *p* < 0.001, *η*_*p*_^2^ = 0.607]. Figure 2B shows the magnitude estimates of pleasantness as a function of compliance. Pleasantness monotonically increased and reached a plateau at 7.56 mm/N (i.e., 0.879 in the horizontal axis in Fig. 2B). The linear trend again explained most of the variance. R^2^ values for fitted functions with 5 N and 20 N exceeded 0.98. This ANOVA also showed a significant main effect of maximum force, with lower force showing higher estimates than higher force [*F*(1, 23) = 5.84, *p* = 0.024, *η*_*p*_^2^ = 0.202] and the interaction between compliance and force [*F*(3.9, 90.5) = 3.85, *p* = 0.007, *η*_*p*_^2^ = 0.143]. The slope of the linear function fit to the averaged data was greater for 20 N (0.264) than 5 N (0.202).

### Supplementary analysis

The measurement of compliance can be affected by the size of the probe and the maximum of the applied force. We conducted a supplementary analysis using compliance of stimuli that was measured with a different probe (flat-end cylindrical probe of 2 cm^2^ area) and a different maximum force (4 N). Nevertheless, the result was highly similar; the linear trend again explained most of the variance (Supplementary Table 1). R^2^ values for fitted functions with 5 N and 20 N exceeded 0.98 in both instruction groups.

**Table 1:** Parameters and goodness of fit of linear functions fit to the data for each participant.

Instruction	Maximum applied force (N)	Slope		Intercept		R^2^	
		Mean	SEM	Mean	SEM	Mean	SEM
Softness	5	0.49	0.06	0.91	0.04	0.90	0.02
	20	0.53	0.06	0.89	0.04	0.93	0.01
Pleasantness	5	0.20	0.03	0.89	0.03	0.77	0.05
	20	0.26	0.04	0.71	0.05	0.78	0.04

### The relationship between softness and pleasantness as a function of compliance

**Figure 3:**
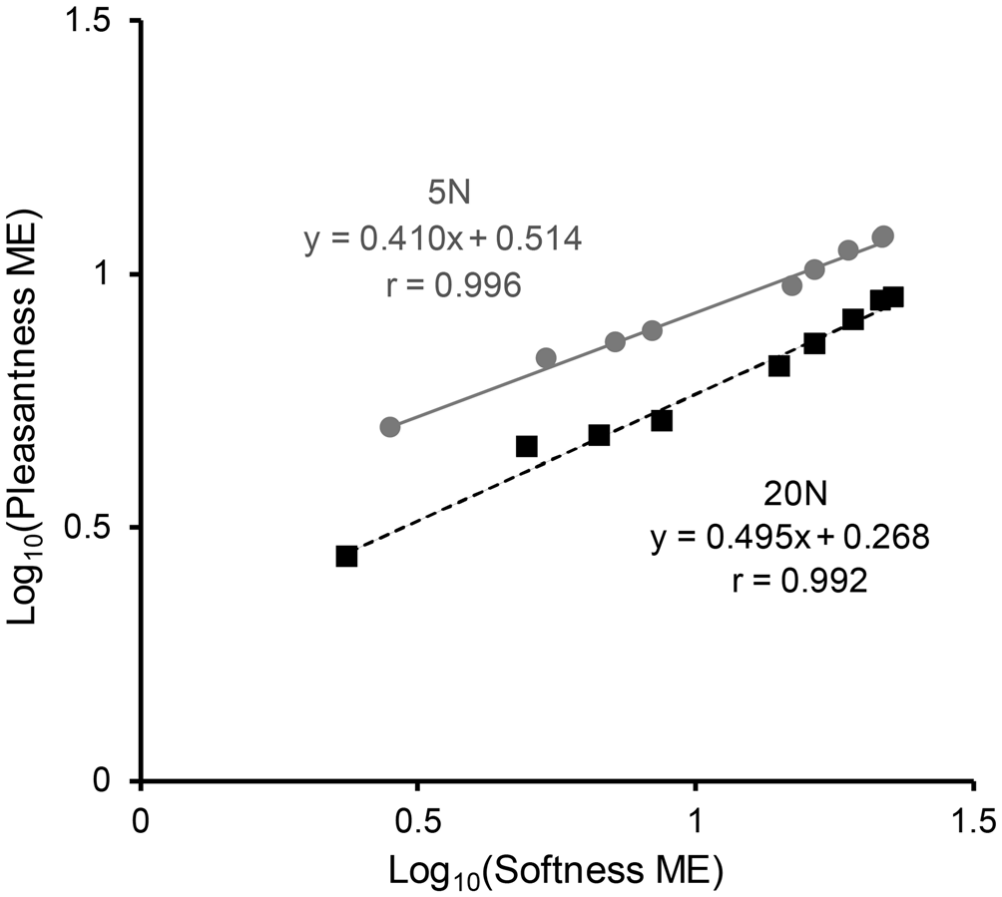
Magnitude estimates of softness and pleasantness. The relationship between log_10_ normalised magnitude estimates (ME) of softness and pleasantness is shown. Each data point indicates mean data for each stimulus.

As both softness and pleasantness increased as a function of compliance, we examined the relationship between the mean magnitude estimates of softness and pleasantness on log_10_–log_10_ scales for two levels of maximum force (Fig. 3). Each dot indicates softness and pleasantness scores for each stimulus. Pearson’s correlation coefficients (r) for the 5 N and 20 N conditions were 0.996 and 0.992, respectively. The mean slope values of the linear function fit to pleasantness estimates as a function of softness were below 1 (0.41 for 5 N and 0.50 for 20 N), indicating that the effect of compliance was smaller for pleasantness than for softness.

To confirm differences in the effect of compliance on magnitude estimates, we fit linear functions of compliance to individual data and statistically compared the slopes between softness and pleasantness (Table 1). The mean coefficients of determination (R^2^) for softness and pleasantness were 0.92 and 0.78, respectively. We performed a two-way ANOVA on slopes with instruction (two levels: softness and pleasantness) as the between-subject variable and applied force as the within-subject dependent variable (two levels: 5 N and 20 N). This analysis revealed significant main effects of force [*F*(1, 46) = 17.88, *p* < 0.001, *η*_*p*_^2^ = 0.28] with 20 N showing a steeper slope than 5 N, and for instruction, with softness showing a steeper slope than pleasantness [*F*(1, 46) = 16.54, *p* < 0.001, *η*_*p*_^2^ = 0.265]. No significant interaction was observed [*F*(1, 46) = 0.88, *p* = 0.354, *η*_*p*_^2^ = 0.019]. The same analysis was conducted with compliance of stimuli that was measured differently (up to 4 N for 2 cm^2^ probe). Nevertheless, the result was highly similar: significant main effects of force [*F*(1, 46) = 15.63, *p* < 0.001, *η*_*p*_^2^ = 0.254] with 20 N showing a steeper slope than 5 N, and for instruction, with softness showing a steeper slope than pleasantness [*F*(1, 46) = 16.58, *p* < 0.001, *η*_*p*_^2^ = 0.265]. No significant interaction was observed [*F*(1, 46) = 0.89, *p* = 0.349, *η*_*p*_^2^ = 0.019] (Supplementary Table 2).

**Table 2:** Pearson’s correlation coefficients of magnitude estimates between the participants.

		Pleasantness				Softness			
		Low force		High force		Low force		High force	
	Force	Mean	SEM	Mean	SEM	Mean	SEM	Mean	SEM
Pleasantness	Low	0.733	0.015	0.747	0.015	0.819	0.012	0.832	0.011
	High	0.747	0.015	0.758	0.012	0.829	0.010	0.840	0.010
Softness	Low	0.819	0.012	0.829	0.010	0.905	0.005	0.924	0.004
	High	0.832	0.011	0.840	0.010	0.924	0.004	0.942	0.003

### The effect of maximum force on softness and pleasantness

Additionally, we compared the effect of force between softness and pleasantness. We defined the effect of maximum force by calculating log_10_ (normalised ME in 5 N/normalised ME in 20 N). While the force effect varied among different levels of compliance, the effect of force on magnitude estimates was greater for pleasantness than for softness at each level of compliance. A two-way ANOVA on the force effect with instruction and compliance as independent variables showed significant main effects of compliance [*F*(4.2, 194.2) = 5.08, *p* = 0.001, *η*_*p*_^2^ = 0.099] and instruction [*F*(1, 46) = 4.75, *p* = 0.034, *η*_*p*_^2^ = 0.094]. No significant interaction was observed [*F*(4.2, 194.2) = 0.57, *p* = 0.697, *η*_*p*_^2^ = 0.012].

### Consistency of magnitude estimates between the participants

As error bars (SEMs) in Fig. 2 indicate, there were greater individual differences in the patterns of magnitude estimates for pleasantness than for softness. In order to further confirm this point, we calculated Pearson’s correlation coefficient (r) of magnitude estimates between each pair of the participants within each condition or between conditions (Table 2). There are two points worth noting. First, the r values for within-condition inter-subject correlation were greater in the softness condition than in the pleasantness condition, indicating that the patterns of magnitude estimates are more similar among the participants in the softness condition than among those in the pleasantness condition. Random permutation tests showed that r values in the softness condition were significantly greater than those in the pleasantness condition at each force level (*p* values < 0.001, with Bonferroni correction). Second, mean r values in correlation between different conditions (e.g., low-force softness vs. low-force pleasantness) were over 0.8, which is consistent with the strong correlation between softness and pleasantness at the group level (Fig. 3).

### Sex differences

We conducted a supplementary analysis to examine the effect of sex on magnitude estimates. We conducted a three-way ANOVA on magnitude estimates in softness with compliance (nine levels) and maximum force (two levels: 5 N or 20 N) as within-subject variables and sex (two levels) as a between-subject variable. However, there was neither a significant main effect of sex [*F*(1, 22) = 1.49, *p* = 0.236, *η*_*p*_^2^ = 0.063] nor significant interaction with sex [*F*(1, 22) = 0.65, *p* = 0.43, *η*_*p*_^2^ = 0.028 for the interaction between force and sex; *F*(1.3, 28.5) = 0.76, *p* = 0.424, *η*_*p*_^2^ = 0.033 for the interaction between compliance and sex; *F*(3.6, 78.2) = 1.36, *p* = 0.259, *η*_*p*_^2^ = 0.058 for the three-way interaction]. The same ANOVA for pleasantness revealed neither main effect of sex [*F*(1, 22) = 1.91, *p* = 0.181, *η*_*p*_^2^ = 0.08] nor interaction with sex [*F*(1, 22) = 0.049, *p* = 0.827, *η*_*p*_^2^ = 0.002 for the interaction between force and sex; *F*(1.5, 34.1) = 1.54, *p* = 0.229, *η*_*p*_^2^ = 0.065 for the interaction between compliance and sex; *F*(4.0, 87.5) = 0.835, *p* = 0.506, *η*_*p*_^2^ = 0.037 for the three-way interaction]. Thus, the effects of sex on magnitude estimates for softness and pleasantness were negligible.

### Additional experiments

The results of the experiment above raised two questions. The first was whether the close relationship between softness and pleasantness can be extended to another set of skin-like stimuli. The second question was whether the participants in the pleasantness-instruction group merely answered magnitude estimates associated with the physical parameter of interest although they experienced highly similar pleasantness across stimuli. We conducted two additional experiments to address the two questions (see Supplementary Information 2 for more details).

The first experiment was identical to the main experiment except for type of stimuli (spherical segments of urethane elastomer covered by a plastic membrane) and number of fingers used (two) (Supplementary Fig. 1). We confirmed that magnitude estimates of pleasantness and softness increased monotonically as a function of compliance. The correlation coefficients between the mean magnitude estimates of softness and pleasantness was above 0.98. Moreover, function for pleasantness was lower than that for softness (*p* values < 0.05, post-hoc pairwise comparisons with Bonferroni correction. See more details in Supplemental Information 2). Random permutation tests showed that *r* values in the softness condition were significantly greater than those in the pleasantness condition at each force level (*p* values < 0.001, with Bonferroni correction). Thus, these results were consistent with the result of the main experiment.

The second experiment was identical to the first additional experiment except for type of stimuli and instruction. Stimuli were spherical segments made of polystyrene foam (2 cm diameter), varying in number from one to five. As the shape and material properties of each sphere were identical in all stimuli, the effect on pleasantness should have been similar across stimuli. The participants who judged softness in the first additional experiment estimated the number of spheres in this experiment, whereas the participants who judged pleasantness in the first additional experiment again evaluated pleasantness in this experiment. Unlike the first additional experiment, the magnitude estimates for pleasantness were highly similar across the stimuli, whereas the estimates for the number of spheres increased monotonically with the number of spheres (Supplementary Fig. 2). One-sample *t* tests on the mean slopes of linear functions fitted to individual data showed significance only in the group for the number of spheres [*t*(11) = 12.05, *p* < 0.001, *d* = 3.479 for the number of spheres; *t*(11) = 1.01, *p* = 0.3 for the pleasantness]. Thus, we confirmed that the reported pleasantness values were not associated with the manipulated physical factor (number of spheres), when such a factor was not related to pleasantness.

## Discussion

The present study had two main findings. First, the magnitude of pleasantness increased as a function of compliance. Second, the slope of the linear function that was fit to the magnitude estimates was steeper for softness than for pleasantness. Individual variability was greater for the pleasantness than for the softness group.

We used pieces of rubber that were made of the same material and minimised tackiness by putting powder on all of them. Thus, it is unlikely that any potential tackiness of the stimuli with high compliance affected our results. We also kept the speed and applied force the same between softness and pleasantness conditions. Therefore, differences in these physical factors should not explain differences between softness and pleasantness conditions. Finally, participants were given different instructions for softness and pleasantness, with no hint of an association between the two. Moreover, we observed different effects of force on softness relative to pleasantness and less inter-individual consistency for pleasantness than for softness. Additional experiments confirmed that magnitude estimates for pleasantness were highly similar among stimuli when the physical factor of interest was not related to pleasantness. Thus, it is highly unlikely that participants in the pleasantness group answered with softness magnitude instead of with pleasantness magnitude. Rather, our results indicated that object compliance contributes to increases in the tactile perception of pleasantness and softness.

One of the main findings of the present study was that pleasantness increased monotonically as a function of compliance and reached a plateau at 7.56 mm/N; only a weak increase of estimates was observed after this compliance value. Among major perceptual dimensions of material properties^23,24^, previous studies investigated associations between discriminative and affective aspects of touch as a function of temperature^28^ and roughness^25–27^. This study extends these previous findings by demonstrating the effect of compliance on pleasantness.

Our findings are consistent with the view that affective touch is related to how physical contact with an object can potentially influence the physiological condition of the self body^27,39^. For example, Mower (1976) showed that when participants placed their right hands into water at different temperatures, their body temperatures changed. Additionally, he found that warmer water felt more pleasant under the condition of hypothermia, whereas colder water felt more pleasant under the condition of hyperthermia^28^. Another study showed that rougher surfaces felt more unpleasant than smoother surfaces, presumably because rough surfaces can potentially damage one’s skin and should be avoided^27^. As with the perception of temperature and roughness, it is possible that a harder object would feel less pleasant than a softer object, because contact with hard objects can potentially cause more physical damage to the human body than does contact with soft objects.

Furthermore, smooth and soft surfaces are often associated with objects that are beneficial. For example, such contact is related to skin-to-skin contact with conspecifics, and contact with one’s partner can decrease unpleasantness related to physical pain^7^ and social pain^8^. In a classic experiment, baby monkeys chose to stay with surrogate mothers wrapped in a soft cloth rather than surrogate mothers made of wires^5^. Thus, it is possible that the softness of our bodies is associated with pleasantness to promote interpersonal communication. As in social grooming among monkeys, tactile pleasantness might contribute to maintaining and promoting social structures in humans^9^.

Previous studies that used affective adjectives examined the factors that contribute to affective touch and compared them to the factors that contribute to discriminative touch^29,31^. For instance, one study compared principal components analyses (PCA) scores from ratings of affective adjectives to PCA scores from ratings of discriminative (“sensory”) adjectives^29^. The component that is associated with valence was not correlated with the discriminative component that includes deformable adjectives (deformable, hard, firm, and elastic), but instead correlated with the “roughness” component. However, this “roughness” factor included the adjective “softness” as well as adjectives for roughness. Likewise, the factor labelled “silken” that included “smooth” and “soft” attributes was correlated with the factor of “pleasantness” in the other study^31^. There are strong correlations between smoothness and pleasantness^25–27^, whereas the present study showed a correlation between softness and pleasantness. The present study is not necessarily contradictory to previous studies; our finding rather complements existing research on pleasantness by controlling factors other than compliance.

We observed larger inter-subject variability in pleasantness than softness, which was confirmed in the additional experiment. This result is consistent with the idea that veridical perception is less necessary for assessing pleasantness than for assessing softness. Unlike discriminative touch, affective touch is subject to fewer such demands, inasmuch as the emotional components relate to subjective and internal states^27^. This speculation could also explain why the slope of the fit for the linear function was shallower for pleasantness than softness, as a slight difference in compliance does not influence affective sensation as much as it influences softness.

The effect of force on magnitude estimates was stronger for pleasantness than for softness in the main experiment. Although consistent with our prediction, this finding was not confirmed in the first additional experiment. This difference seems to be derived from the result of the softness group; the force effect was negligible for the main experiment, whereas there was interaction between force effect and compliance in the additional experiment. One reason for the difference was the type of stimuli. Because of the size of stimuli, fewer fingers were stimulated in the additional experiment. Because the area of contact between skin and object was smaller in the additional experiment, the maximum force (up to 20 N) may provide more differentiation clues than 5 N did. Thus, further studies are necessary to test the perceptual constancy of softness against applied force.

The present study has several limitations. Although previous studies on softness and roughness typically used one type of stimuli^17,18,26,27,34,41,42^, we used two types of rubber stimuli to confirm consistency for two reasons. First, we were particularly interested in the pleasantness evoked by touching human skin, and contact with soft rubber can feel similar to contact with human skin. Second, our stimuli were similar to the stimuli used in other studies in that their surfaces were deformable^19,34,43,44^. However, as in studies in psychology and neuroscience^45,46^, it is not clear to what extent the result with one type of stimuli can be generalised to other types of stimuli. For example, objects with rigid surfaces, such as spring cells, may not feel pleasant at all^19,33^. Thus, it is worth testing more sets of stimuli to model them as a random-effect design in order to test the extent of generalisation to deformable objects. Further, since we matched the maximum force across stimuli, the duration of stimulation was not identical for all stimuli. Softer stimuli could be presented for a longer duration, as they take more indentation before reaching the specified force. Though longer contact with a hard object is unlikely to increase pleasantness, this point should be experimentally confirmed in future studies. Finally, the present study adopted passive stimulation to control the speed and force of stimuli. Thus, it is important to test if the same effect of compliance on pleasantness can occur in active touch.

Despite such limitations, our findings can contribute to the development of a comprehensive psychophysical model for the tactile sensation of pleasantness, which will be particularly valuable to the field of product design and marketing. Our findings may allow us to select an appropriate range of compliance to control the pleasantness experienced when making contact with an object. For instance, designers should consider how we typically interact with such objects, because the maximum force applied by the object alters the magnitude of pleasantness. In conjunction with previous findings on roughness^25–27^ and temperature^28^, the results from the present study constitute initial steps toward the construction of a psychophysical model of tactile pleasantness. In the future, we should consider how roughness, softness, and temperature interact with each other to cause pleasantness.

In conclusion, the present study investigated patterns of pleasantness as a function of object compliance and compared them to those for softness using skin-like rubber stimuli. Although magnitude estimates of both softness and pleasantness showed highly similar patterns, the effect of compliance between softness and pleasantness differed in magnitude and inter-subject variability. These results indicate that object compliance is a critical physical determinant for tactile pleasantness in rubber-like stimuli, whereas the effect on pleasantness varies among individuals. In conjunction with previous findings, this result highlights the contribution made by mechanoreceptors in the hand to affective touch.

## Methods

### Participants

Participants in the present study included 48 right-handed volunteers (24 males and 24 females). Their mean age was 23.6 years (ranging from 18 to 35 years old). Participants were recruited via flyers and received 10 Singaporean dollars (SGD) for their participation. All participants were free from finger and/or hand injuries and were right-handed according to the Fazio Handedness Inventory^47^, which is a modified version of the Edinburgh Handedness Inventory^48^. All participants provided written informed consent prior to beginning the experiment. The study protocol was approved by the Institutional Review Board at Nanyang Technological University Singapore (IRB-2018–07-013). All methods were carried out in accordance with the approved guidelines.

### Stimulus

We used nine stimuli made of polyurethane rubber, which is not carcinogenic (Katō Tech Co. Ltd., Kyoto, Japan; Fig. 1A). We chose polyurethane rubber because the surface is deformable like skin and similar to stimuli used in previous studies^19,33,34,44^. Each stimulus consisted of a plastic box (9.5 cm length by 9.5 cm width by 2.5 cm depth) filled with polyurethane rubber (Katō Tech Co. Ltd., Kyoto, Japan). One side of each box was open to allow participants to touch the flat surface of the material directly. Baby powder (corn starch) was put on each surface to minimize the tackiness of the rubber. The compliance of each stimulus was measured with a compression tester (KES-G5, Katō Tech Co. Ltd., Kyoto, Japan). In this measurement, each stimulus was placed on the platform of the tester and a flat-end cylindrical probe of 1 cm^2^ area was brought down to indent the surface of each stimulus centrally at a constant speed of 0.5 mm/s until the applied force reached around 100-g force. One-hundred-gram force per 1 cm^2^ is close to the condition when three fingers were stimulated at 5 N [e.g., 5 N / (over 2 cm^2^ per finger × 3 fingers) ≈ 85-g force per 1 cm^2^]. Figure 1 shows representative data of the relationship between the indentation and the applied force. We fit a linear function of the applied force to each repetition of each stimulus and calculated the average of the fitted slopes across repetitions. Consequently, the compliances of the stimuli were 0.13, 0.45, 0.77, 1.06, 2.36, 3.52, 5.54, 7.56, and 10.53 mm/N. For example, the compliance of the dorsum of the hand (the skin over the first dorsal interosseous of 7 females, 20–30 years old) ranged from 8.1 to 11 mm/N when it was pressed up to 0.5 N.

### Stimulus presentation

A customized apparatus was used to stimulate each participant’s fingers. We developed a stimulator by modifying an industrial device that tests the properties of springs (Model SHR III-5 SK Aikoh Engineering, Japan; Fig. 1C). Thus, speed and maximum applied force were controlled to measure the compliance of the stimuli. We modified gloves so that participants’ index, middle, and ring fingers could touch stimuli directly and glued a sheet of Velcro to the dorsal part of the gloves. Participants wore gloves on their right hands and placed their hands with their fingers facing upward (Fig. 1C). Styrofoam and a roll of felt supported the position of their right hands to allow participants to relax their fingers during the experiment. Each stimulus was moved down to press against participants’ fingers at 5 cm/s. Once the force applied to their fingers reached either 5 N or 20 N, the device moved back to its initial position. We ensured that participants did not feel pain during the experiment.

### Design and procedures

We adopted an experimental design similar to that of our previous study^27^ with one between-subject and two within-subject variables. We treated compliance (nine levels) and maximum applied force (two levels: 5 N and 20 N) as within-subject variables, whereas instruction (judging softness and pleasantness) was designated as the between-subject variable to avoid the participant treating softness and pleasantness as identical. The ratio of biological sex was matched between the two groups, and their ages were matched as well [*t*(46) = 1.28, *p* > 0.2]. The order in which the nine stimuli and two forces were presented was pseudo-randomly determined for each of four repetitions per participant, such that each force/stimulus combination was presented once within each repetition. The first repetition was considered practice and was excluded from statistical analysis.

Blindfolded participants were seated in a chair with the apparatus to their right. An absolute magnitude-estimation procedure was used to estimate either the softness or pleasantness of each stimulus^49^. In one group, participants were instructed to choose a number that best matched the perceived softness of the stimulus. In the other group, participants were instructed to choose a number that best matched the magnitude of pleasantness associated with each stimulus. Participants were allowed to use any number (decimal, fraction, or whole number), as long as the number was above zero. Neither modulus nor standard was used. Participants were instructed to keep the same posture throughout the experiment. During the practice repetition, all participants were familiarized with the magnitude-estimation procedure. Each session lasted approximately 45 min. After the experiment ended, the experimenter confirmed that none of the participants experienced any painful sensations during the experiment.

### Analysis

We used the conventional procedure to analyse magnitude estimate scores^21,27^. The data averaged across three repetitions were normalized within each perceptual condition to eliminate possible biases due to the participants' use of different number ranges. This normalization procedure was performed by dividing each data point by the participant’s mean and then multiplying that by the grand mean for the group (softness or pleasantness). Finally, to provide a more normal distribution of the magnitude estimates^50^, the scores were logarithmically transformed (base 10). These transformed data were used for data analyses. The Greenhouse–Geisser correction was applied to adjust for the lack of sphericity in repeated-measures ANOVA.

Softness and pleasantness are qualitatively different perceptual dimensions. Thus, as in our previous study^27^, we did not initially directly compare the magnitude estimates for softness and pleasantness within the same analysis. We began by analysing the data for each instruction condition separately and subsequently compared softness and pleasantness directly in terms of the effects of compliance and applied force. We used IBM SPSS Statistics (version 23.0, IBM Corp., Armonk, NY, U.S.) for these analyses.

In order to examine consistency of magnitude estimates among the participants, we calculated Pearson’s correlation coefficients (r) between them. For each condition (e.g., low-force softness condition), we calculated r values between each possible pair of the participants in the same condition and obtained 276 r values (_24_C_2_). To compare different conditions, we calculated r values for all combinations of the participants (24 × 24 = 576). In order to statistically evaluate the difference in r values between the softness and pleasantness groups in each force level, we conducted random permutation tests with MATLAB (version 2017b, The MathWorks, Inc., Natick, MA, U.S.). More specifically, we randomly shuffled the participants between the two instruction groups and calculated the r values for inter-subject correlation, repeating this procedure 10,000 times. We calculated the p value by counting the number of permutations with equal or higher accuracy than the accuracy of the original result and corrected it for multiple comparisons (using Bonferroni correction)^44^.

## Supplementary information

Supplementary information
