# Supplementary material for: Tactile perception of pleasantness in relation to perceived softness
Source: Sci Rep. 2020 Jul 7;10:11189. doi: 10.1038/s41598-020-68034-x (PMC7341757; doi:10.1038/s41598-020-68034-x)
Supplement: Supplementary file 1 — Supplementary information [file 41598_2020_68034_MOESM1_ESM.docx]

**Supplementary Information**

Tactile Perception of Pleasantness in Relation to Perceived Softness

Achille Pasqualotto^1, 2^, Megan Ng^2^, Zheng Yee Tan^2^, and Ryo Kitada^2^

1. School of Psychology, University of Nottingham Malaysia, Malaysia
2. Division of Psychology, School of Social Sciences, Nanyang Technological University, Singapore

**^*^Corresponding author:**

Ryo Kitada

School of Social Sciences, Nanyang Technological University, 48 Nanyang Avenue, 639818, Singapore. Tel: +65 6316 8935. E-mail: [ryokitada@ntu.edu.sg](mailto:ryokitada@ntu.edu.sg)

**Supplementary Information 1**

Measurement of compliance can be influenced by the size of the stimulus, the maximum force, and the size of a probe. In order to confirm that our result was not affected by these factors, we measured compliance of stimuli with the maximum force of 4 N and the flat-end cylindrical probe of 2 cm^2^ area. Consequently, compliance values (per 1 cm^2^) for the stimuli were 0.12, 0.54, 0.92, 1.29, 2.73, 4.03, 5.75, 7.14, and 8.51 mm/N.

**Supplementary Table 1.** **Parameters and goodness of fit of linear functions fit to the averaged data (with the other definition of compliance)**

| Instruction | Maximum  applied force | Slope | Intercept | R^2^ |
| --- | --- | --- | --- | --- |
|  |  | Mean | Mean | Mean |
| Softness | 5 N | 0.51 | 0.89 | 0.99 |
|  | 20 N | 0.55 | 0.86 | 0.99 |
| Pleasantness | 5 N | 0.21 | 0.88 | 0.99 |
|  | 20 N | 0.27 | 0.70 | 0.98 |

**Supplementary Table 2.** **Parameters and goodness of fit of linear functions fit to each participant’s data (with the other definition of compliance)**

| Instruction | Maximum  applied force | Slope | | Intercept | | R^2^ | |
| --- | --- | --- | --- | --- | --- | --- | --- |
|  |  | Mean | SEM | Mean | SEM | Mean | SEM |
| Softness | 5 N | 0.51 | 0.06 | 0.90 | 0.04 | 0.90 | 0.02 |
|  | 20 N | 0.55 | 0.06 | 0.87 | 0.04 | 0.93 | 0.01 |
| Pleasantness | 5 N | 0.21 | 0.04 | 0.88 | 0.03 | 0.77 | 0.05 |
|  | 20 N | 0.27 | 0.04 | 0.70 | 0.05 | 0.78 | 0.05 |

**Supplementary Information 2: Additional experiments**

We conducted two additional experiments to address two questions. The first question was whether the close relationship between softness and pleasantness (i.e., strong correlation) could be applied to other stimuli with deformable surfaces (like human skin). The second question was whether participants may have merely reported values associated with the physical characteristic of interest. In the two experiments, 24 right-handed volunteers (16 women and 8 men, 22.9 ± 2.1 years old, mean ± SD) participated. The number was determined by the effect size in the main experiment (i.e., the effect of compliance on pleasantness) and the previous study^27^. As in the main experiment, participants were assigned to one of the two instruction groups. The ratio of biological sex was matched between the two groups (8 women and 4 men for each group), and their ages were matched as well [*t*(22) = 0.3, *p* > 0.7].

**Additional experiment 1**

***Methods***

The experiment was identical to the main experiment except for the stimuli and the number of fingers stimulated. We used seven spherical segments made of urethane elastomer (Bioskin; Beaulax Co., Ltd., Tokyo, Japan). The compliances of the stimuli were 0.67, 1.02, 1.16, 1.96, 3.08, 5.20, and 7.49 mm/N. The critical differences from the stimuli used in the main experiment were twofold. First, these stimuli were covered with a plastic membrane to control the characteristics of the surface. Second, stimuli size and shape were different from the stimulus set in the main experiment (5-cm diameter base × 1.3-cm height). As a result, the quality of sensation was different from the stimulus set for the main experiment. These stimuli were placed on an identical plastic base (Supplementary Figure 1C). Due to the smaller size of the stimuli, we stimulated the right index and middle fingers of the participants. The other procedure and analysis were identical to the main experiment.

***Results***

Supplementary Figure 1 shows the result of the additional experiment 1. Magnitude estimates monotonically increased with the increase of compliance in both instructions. R^2^ values (the coefficient of determination) for fitted linear functions with 5 N and 20 N exceeded 0.93 in both instructions, indicating that the linear trend explained most of the variance in the averaged data.





**Supplementary Figure 1. Additional Experiment 1**

**A.** Mean log_10_ normalised magnitude estimates (ME) of pleasantness as a function of compliance for low and high maximum force. **B.** Mean log_10_ normalised ME of softness as a function of compliance for low and high maximum force. Each data point indicates mean ± SEM of 12 participants in each group. Note that two groups of participants evaluated softness and pleasantness separately. **C.** The experimental setup was the same as the main experiment except for stimuli and stimulated fingers (index and middle fingers). **D.** The relationship between log_10_ normalised magnitude estimates (ME) of softness and pleasantness is shown. Each data point indicates mean data for each stimulus.

We compared mean slopes of the fitted linear functions to individual data. One-sample *t* tests (with Bonferroni correction) showed that slopes in each condition were greater than zero [*t*(11) = 4.0, *p* = 0.008, *d* = 1.155 for 5 N in the pleasantness instruction; *t*(11) = 5.19, *p* = 0.001, *d* = 1.499 for 20 N in the pleasantness instruction; *t*(11) = 6.74, *p* < 0.001, *d* = 1.946 for 5 N in the softness instruction; *t*(11) = 7.94, *p* < 0.001, *d* = 2.291 for 20 N in the pleasantness instruction]. We then performed a two-way ANOVA on slopes with instruction (two levels: softness and pleasantness) as the between-subject variable and applied force as the within-subject dependent variable (two levels: 5 N and 20 N). This analysis revealed significant main effects of force [*F*(1, 22) = 12.0, *p* = 0.002, *η_p_^2^* = 0.353] and of instruction [*F*(1, 22) = 10.0, *p* = 0.005, *η_p_^2^* = 0.313], as well as a significant interaction [*F*(1, 22) = 4.35, *p* = 0.049, *η_p_^2^* = 0.165]. Post-hoc pairwise comparisons with Bonferroni correction revealed that the slope for softness was steeper than the slopes for pleasantness at each force level (*p* values < 0.05).

As in the main experiment, we calculated Pearson’s correlation coefficient (r) of magnitude estimates between each pair of the participants within each condition or between conditions (Supplementary Table 3). The results were highly similar to the main experiment. First, the *r* values for within-condition inter-subject correlation were greater in the softness condition than in the pleasantness condition. Random permutation tests confirmed greater *r* values in the softness condition than those in the pleasantness condition at each force level (*p* values < 0.001, with Bonferroni correction). Second, mean *r* values in correlation between different conditions (e.g., low-force softness vs. low-force pleasantness) were over 0.7.

**Supplementary Table 3. Pearson’s correlation coefficients of magnitude estimates between the participants**

|  |  | Pleasantness | | | | Softness | | | |
| --- | --- | --- | --- | --- | --- | --- | --- | --- | --- |
|  |  | Low force | | High force | | Low force | | High force | |
|  | Force | Mean | SEM | Mean | SEM | Mean | SEM | Mean | SEM |
| Pleasantness | Low | 0.576 | 0.074 | 0.671 | 0.038 | 0.741 | 0.041 | 0.757 | 0.039 |
|  | High | 0.671 | 0.038 | 0.728 | 0.036 | 0.817 | 0.021 | 0.825 | 0.021 |
| Softness | Low | 0.741 | 0.041 | 0.817 | 0.021 | 0.942 | 0.005 | 0.941 | 0.004 |
|  | High | 0.757 | 0.039 | 0.825 | 0.021 | 0.941 | 0.004 | 0.960 | 0.005 |

Finally, we examined the effect of maximum force. A two-way ANOVA on the force effect with instruction and compliance as independent variables showed a significant main effect of compliance [*F*(2.9, 63.6) = 8.49, *p* < 0.001, *η_p_^2^* = 0.278] and a significant interaction with instruction [*F*(2.9, 63.6) = 4.19, *p* = 0.01, *η_p_^2^* = 0.16]. No significant main effect of instruction was observed [*F*(1, 22) = 1.33, *p* = 0.262, *η_p_^2^* = 0.057]. However, none of the two-sample *t* tests (with Bonferroni correction) on the force effect at each compliance level showed significant difference between the two instructions.

Collectively, we confirmed that compliance is a critical determinant for pleasantness using another set of deformable stimuli. Moreover, slopes of the fitted function were greater for softness than for pleasantness, whereas inter-subject correlation was lower for the softness than for the pleasantness condition.

**Additional experiment 2**

***Methods***

The participants completed this experiment immediately following the first additional experiment. This experimental design was identical to the main experiment except for the following three points. First, stimuli were spherical segments made of polystyrene foam (2-cm diameter base × 1-cm height) that were placed on a plastic base (Supplementary Figure 2). The number of segments placed on each base varied from one to five, resulting in five stimuli. Second, the instruction for the pleasantness-instruction group was identical to that in the main experiment, whereas the participants in the softness instruction group were asked to conduct the magnitude estimation for the number of spherical segments. Third, the maximum force was 5 N, because one participant in a pilot experiment experienced pain in the 20 N stimulation.

If the participants answered values of pleasantness based on physical characteristics that were experimentally varied, then we could expect that the reported pleasantness would increase as a function of the number of spherical segments. By contrast, if the participants were really engaged in the evaluation of pleasantness, then the magnitude estimates should be highly similar across the stimuli, given that contact with the same material would not change the perceived pleasantness.

***Results***

Supplementary Figure 2 shows the result of the additional experiment 2. Magnitude estimates for the number of spherical segments monotonically increased with the number of segments, whereas magnitude estimates for pleasantness were highly similar across the stimuli. R^2^ values (the coefficient of determination) for fitted linear functions were 0.994 for the number-of-sphere group and 0.391 for the pleasantness group. A one-way ANOVA (five levels of the number of segments) on magnitude estimates of pleasantness revealed no significant main effect [*F*(2.3, 25.0) = 1.13, *p* = 0.344, *η_p_^2^* = 0.093]. By contrast, the same ANOVA on estimates of the number of segments showed a significant main effect [*F*(2.0, 22.5) = 73.91, *p* < 0.001, *η_p_^2^* = 0.87].





**Supplementary Figure 2. Additional Experiment 2**

**A.** Mean log_10_ normalised magnitude estimates (ME) of pleasantness as a function of

compliance. The mean slope of the fitted function was not significantly different from zero (p = 0.3). **B.** The mean log_10_ normalised ME of pleasantness as a function of compliance. The mean slope of the fitted function was significantly greater than zero (*p* < 0.001). Each data point indicates mean ± SEM of 12 participants in each group. **C.** The experimental setup was the same as the main experiment except for type of stimuli, number of fingers, and maximum force used (only 5 N). Stimuli contained 1-5 spherical segments. **D.** The relationship between log_10_ normalised magnitude estimates (ME) of pleasantness and number of segments is shown. Each data point indicates mean data for each stimulus.

One-sample *t* tests showed that mean slopes of the fitted function were not significantly different from zero in the pleasantness group [*t*(11) = 1.01, *p* = 0.3], although the same test was significant in the number-of-sphere group [*t*(11) = 12.05, *p* < 0.001, *d* = 3.479]. Finally, a two-sample *t* test showed that mean slope value for the number-of-segment group was significantly greater than for the pleasantness group [*t*(22) = 8.21, *p* < 0.001, *d* = 3.353].

Taken together, the result demonstrates that the magnitude estimates of pleasantness do not change when the physical factor is unlikely to be associated with affect. This result is contradictory to the possibility that the participants reported the magnitude estimates of pleasantness using a parameter of interest even though they experienced negligible difference in pleasantness.
